# Supplementary material for: Pontocerebellar hypoplasia linked mutations of the deadenylase Target of EGR1 (TOE1) impair thermal stability, ribonuclease activity, and oligomerization
Source: J Biol Chem. 2026 May 23;302(7):113186. doi: 10.1016/j.jbc.2026.113186 (PMC13316410; doi:10.1016/j.jbc.2026.113186)
Supplement: Supplementary Material [file mmc1.docx]

**SUPPORTING INFORMATION**

**TABLES**

**Table S1: Plasmids used in this work**

| **Plasmid Backbone** | **Insert** | **Source** |
| --- | --- | --- |
| pHis2 | WT  D64A, E66A  R73S  A103T  F148Y  V173G  E220K  F239S  R253W  S304L  H319Q  H319Y  S496F | Original cDNA synthesis/mutagenesis: Genscript Subcloning: In-house |
| pCDNA3.1 | HA - TOE1 | Genscript |
|  | HA - TOE1 (F148Y) | Subcloned in house. |
|  | MYC- TOE1 |  |
|  | MYC- TOE1 (F148Y) |  |

**Table S2: Synthetic RNA substrates used in this work**

| **RNA Substrate** | **Sequence** | **Source** |
| --- | --- | --- |
| Substrate with poly(A) tail | 5'(FL)-CAAGAGAAUCCUCUCUAAAUAAAAAAAAAAAAAAAAAAAA | DharmaCon |
| Substrate with no poly(A) tail  (Used for RNA ladder) | 5'(FL)-CAAGAGAAUCCUCUCUAAAU | DharmaCon |
| Partial stem of substrate with no poly(A) tail  (Used for RNA ladder) | 5'(FL)-CAAGAGAAUCCU | DharmaCon |
| 2-mer of the substrate  (Used for RNA ladder) | 5'(FL)-CA | DharmaCon |
| Substrate with poly(A) tail (3'-end labelled) | CAAGAGAAUCCUCUCUAAAUAAAAAAAAAAAAAAAAAAAA-(FAM)3' | Genscript |

**Table S3. Deadenylation and exonuclease completion of TOE1 variants.** *Calculated percentage of substrate deadenylated at 10 minutes or percentage of substrate degraded at 30 and 60 minutes for each reported PCH mutation. Replicates (N) are from individual protein preps. Standard deviation and p-value based on a One-Way Anova are reported.*

|  |  | **Completed Deadenylation**  **at 10 Minutes** | | | **Completed Exonuclease**  **at 30 Minutes** | | | **Completed Exonuclease**  **at 60 Minutes** | | |
| --- | --- | --- | --- | --- | --- | --- | --- | --- | --- | --- |
|  |  |  |  |  |  |  |  |  |  |  |
|  | **N** | **Mean (%)** | **Std. Dev.** | **p-value** | **Mean (%)** | **Std. Dev.** | **p-value** | **Mean (%)** | **Std. Dev.** | **p-value** |
| **WT** | 12 | 75.08 | 15.52 | N/A | 60.42 | 19.51 | N/A | 69.75 | 11.89 | N/A |
| **R73S** | 3 | 42.00 | 19.29 | 0.0480 | 2.33 | 2.52 | <0.0001 | 10.67 | 11.72 | <0.0001 |
| **A103T** | 3 | 26.33 | 13.32 | 0.0010 | 8.33 | 2.52 | 0.0003 | 26.67 | 11.50 | 0.0023 |
| **F148Y** | 3 | 26.67 | 32.13 | 0.0011 | 2.00 | 3.46 | <0.0001 | 3.67 | 6.35 | <0.0001 |
| **V173G** | 3 | 78.67 | 5.69 | >0.9999 | 21.67 | 8.51 | 0.0094 | 26.33 | 9.07 | 0.0021 |
| **E220K** | 3 | 78.33 | 9.02 | >0.9999 | 52.67 | 25.74 | 0.9978 | 55.00 | 17.09 | 0.8067 |
| **F239S** | 3 | 45.67 | 39.02 | 0.1057 | 1.33 | 2.31 | <0.0001 | 3.00 | 3.61 | <0.0001 |
| **R253W** | 3 | 80.33 | 7.23 | >0.9999 | 13.33 | 14.98 | 0.0010 | 24.67 | 20.03 | 0.0013 |
| **S304L** | 3 | 76.67 | 7.23 | >0.9999 | 8.33 | 6.11 | 0.0003 | 17.33 | 7.64 | 0.0002 |
| **H319Q** | 4 | 75.75 | 12.84 | >0.9999 | 14.50 | 18.50 | 0.0003 | 26.50 | 26.70 | 0.0005 |
| **H319Y** | 3 | 82.67 | 4.51 | 0.9986 | 22.00 | 22.91 | 0.0102 | 36.33 | 30.14 | 0.0287 |
| **S496F** | 3 | 84.00 | 2.00 | 0.9945 | 59.33 | 21.55 | >0.9999 | 57.00 | 22.11 | 0.9070 |

**Table S4. Summary table of thermal stability data.**

*Calculated average melt temperature for each PCH mutation. Replicates (N) are from individual protein preps. Standard deviation and p-value based on a One-Way Anova are reported.*

|  | **Mean T_m_ (°C)** | **Std. Dev. (°C)** | **N** | **p-value** |
| --- | --- | --- | --- | --- |
| **WT** | 53.54 | 0.70 | 4 | N/A |
| **D64A,E66A** | 57.11 | 0.32 | 3 | <0.0001 |
| **R73S** | 52.58 | 0.64 | 3 | 0.1014 |
| **A103T** | 48.14 | 0.46 | 3 | <0.0001 |
| **F148Y** | 48.14 | 0.56 | 3 | <0.0001 |
| **V173G** | 45.69 | 0.50 | 3 | <0.0001 |
| **E220K** | 51.39 | 0.49 | 3 | <0.0001 |
| **F239S** | 51.79 | 0.24 | 3 | 0.0004 |
| **R253W** | 53.77 | 0.40 | 3 | 0.9987 |
| **S304L** | 52.57 | 0.26 | 3 | 0.0956 |
| **H319Q** | 51.69 | 0.61 | 3 | 0.0002 |
| **H319Y** | 51.51 | 0.20 | 3 | <0.0001 |
| **S496F** | 51.70 | 0.14 | 3 | 0.0002 |

**FIGURES**

**
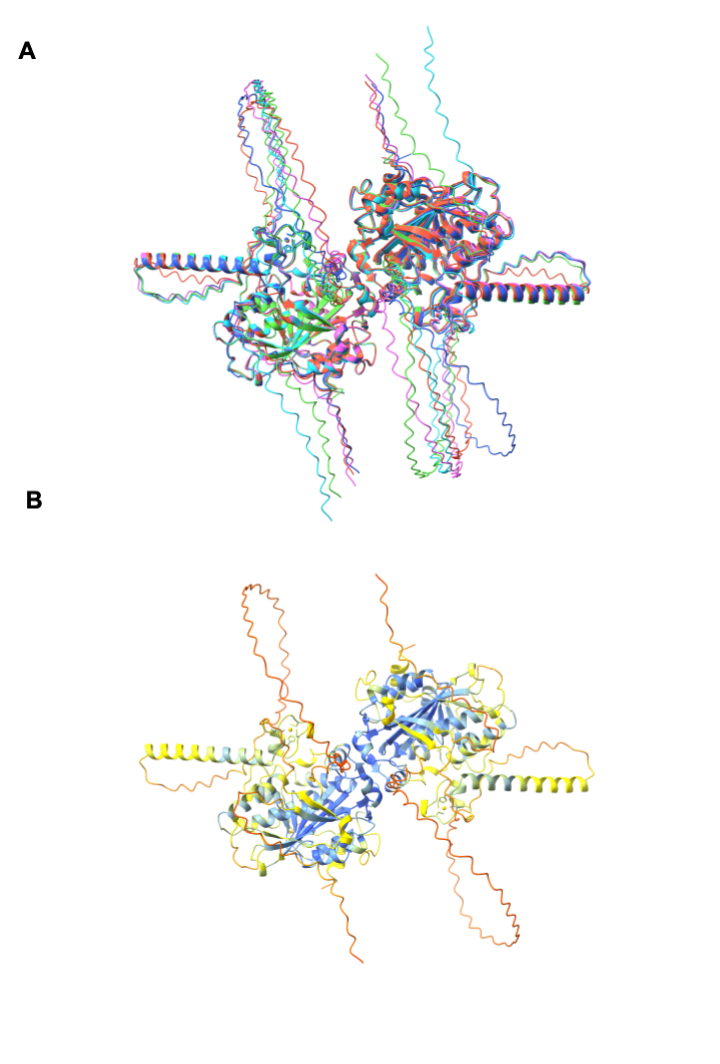
**

**Figure S1: AlphaFold models of TOE1 dimer.** *A) The top 5 models for the TOE1 dimer modeled using AlphaFold3 are shown in an overlay. B) The best AlphaFold model is colored by pLDDT values, using AlphaFold defaults.*

**
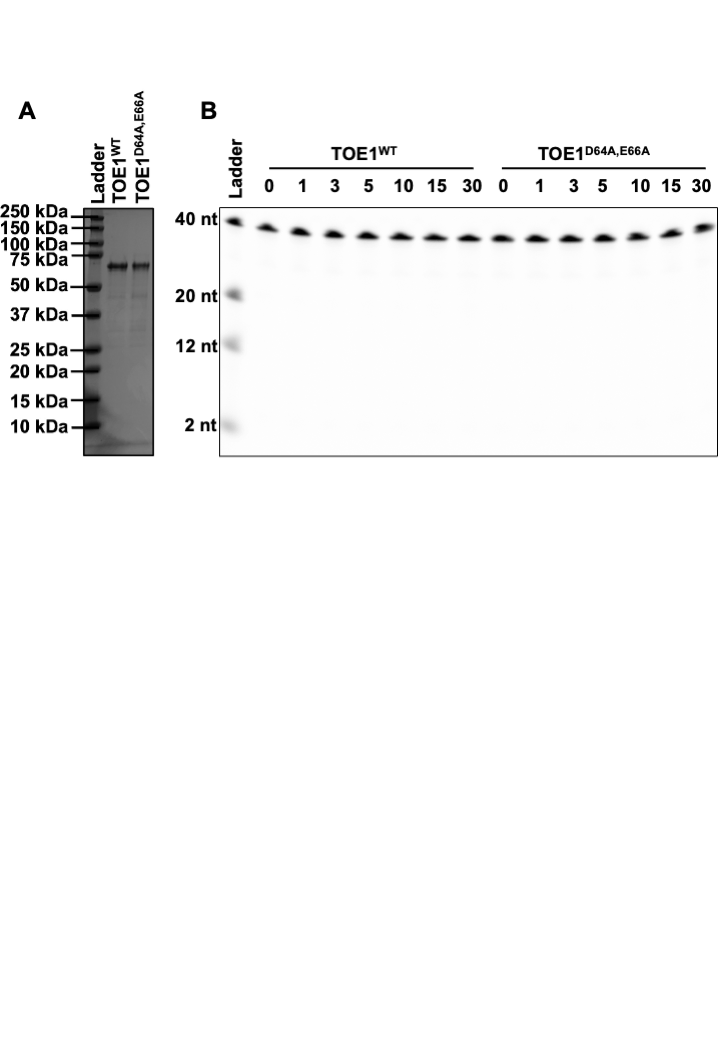
**

**Figure S2: Purification of recombinant TOE1.** *A) Representative SDS-PAGE gel of purified TOE1^WT^ and TOE1^D64A,E66A^. B) TOE1 (250 nM) was assayed for cleavage of a 3'-FAM labeled RNA substrate (500 nM) and was unable to cleave the RNA when the 3'-end is labelled with a fluorophore.*

**
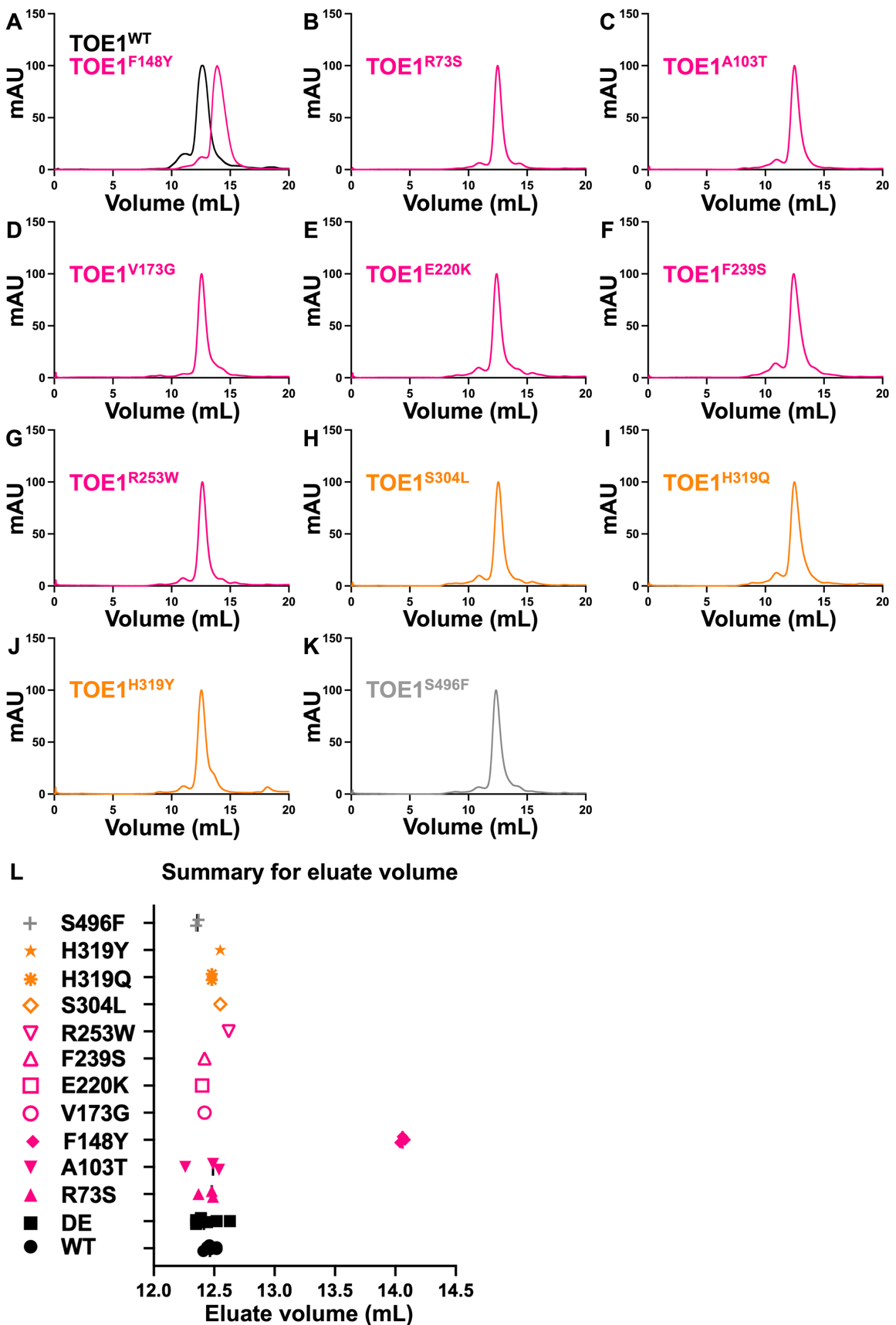
**

**Figure S3: S200 gel filtration traces for PCH7-linked TOE1 variants.** *Profiles of size exclusion chromatographs, normalized to the highest absorbance for each run.* *A) F148Y and WT replicate, B) R73S C) A103T, D) V173G, E) E200K, F) F239S, G) R253W, H) S304L, I) H319Q, J) H319Y, K) S496F, L) Summary of the peak eluate volume for each PCH7-variant measurement. Each datapoint is an independent protein purification.*

**
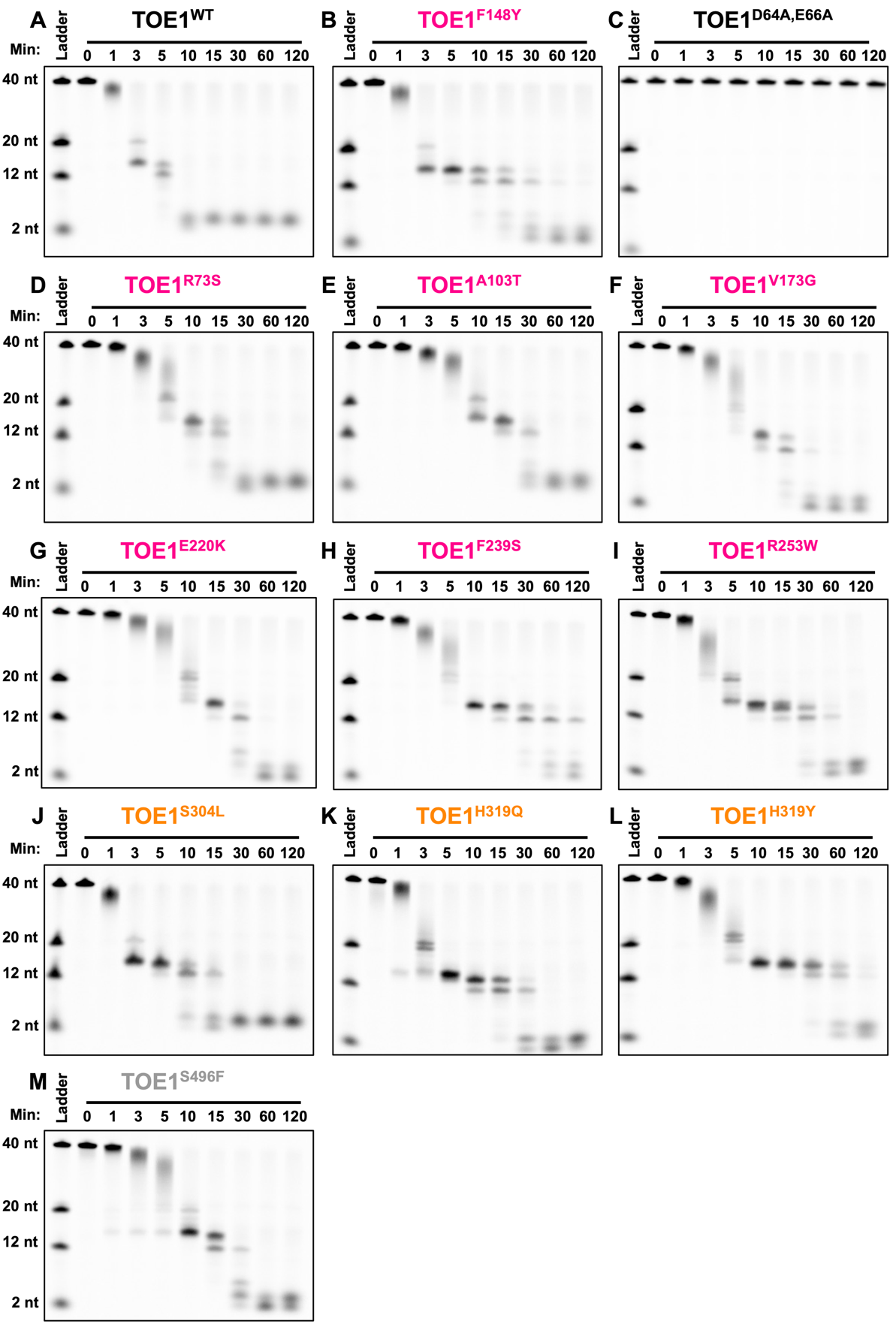
**

**Figure S4: Ribonuclease assays of eluates from the gel filtration for tested variants.** *TOE1 (250 nM) was assayed for cleavage of 5'-fluorescently labeled RNA substrate (500 nM). A) WT, B) F148Y, C) D64A,E66A, D), E) R73S, F) A103T, G) V173G, H) E220K, I) R253W, J) S304L, K) H319Q, L) H319Y. and M) S496F.*
