# Supplementary material for: Pontocerebellar hypoplasia linked mutations of the deadenylase Target of EGR1 (TOE1) impair thermal stability, ribonuclease activity, and oligomerization
Source: J Biol Chem. 2026 May 23;302(7):113186. doi: 10.1016/j.jbc.2026.113186 (PMC13316410; doi:10.1016/j.jbc.2026.113186)
Supplement: Supplementary Material [file mmc2.pptx]

## Slide 1
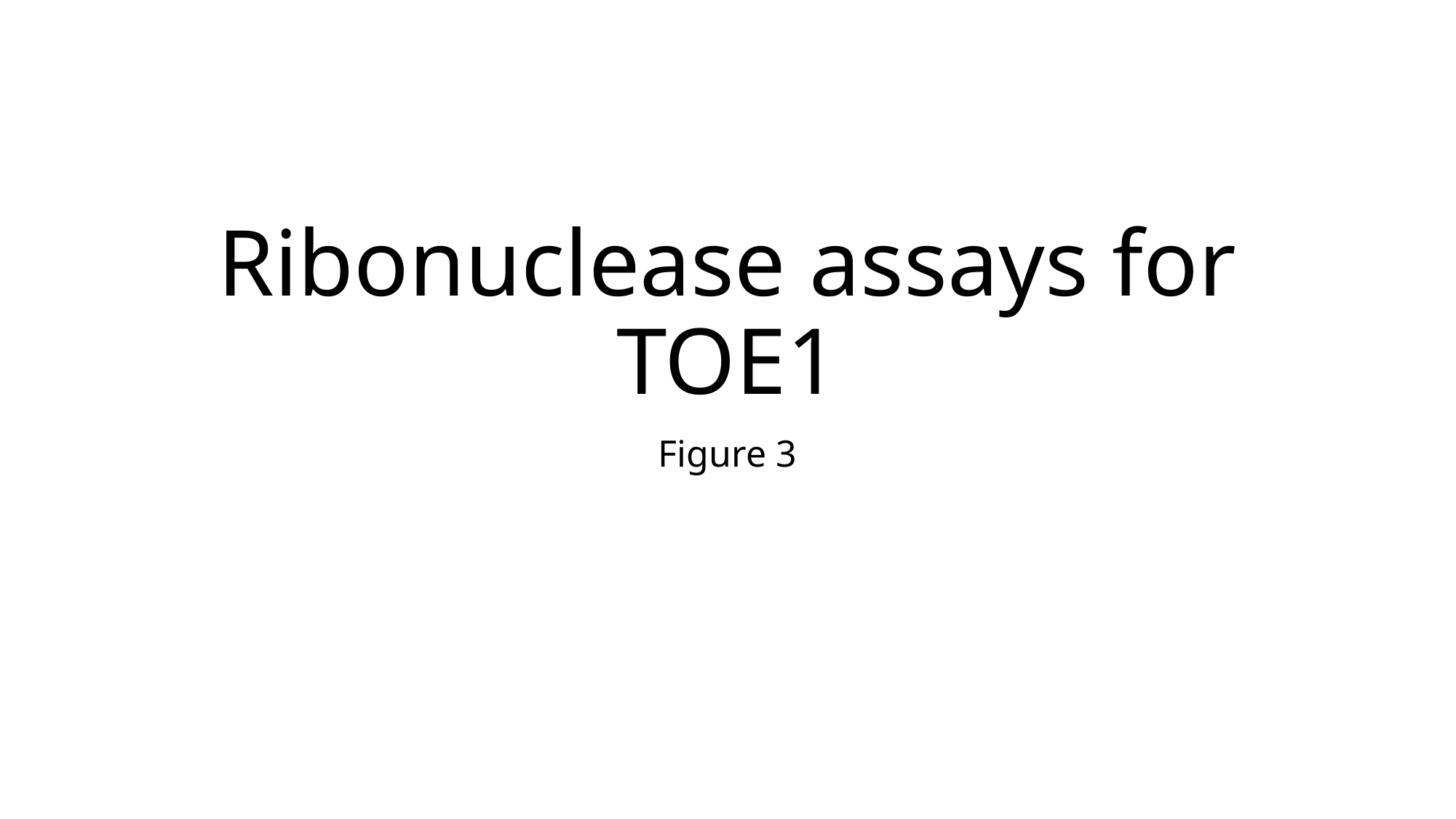

# Ribonuclease assays for TOE1
Figure 3

## Slide 2
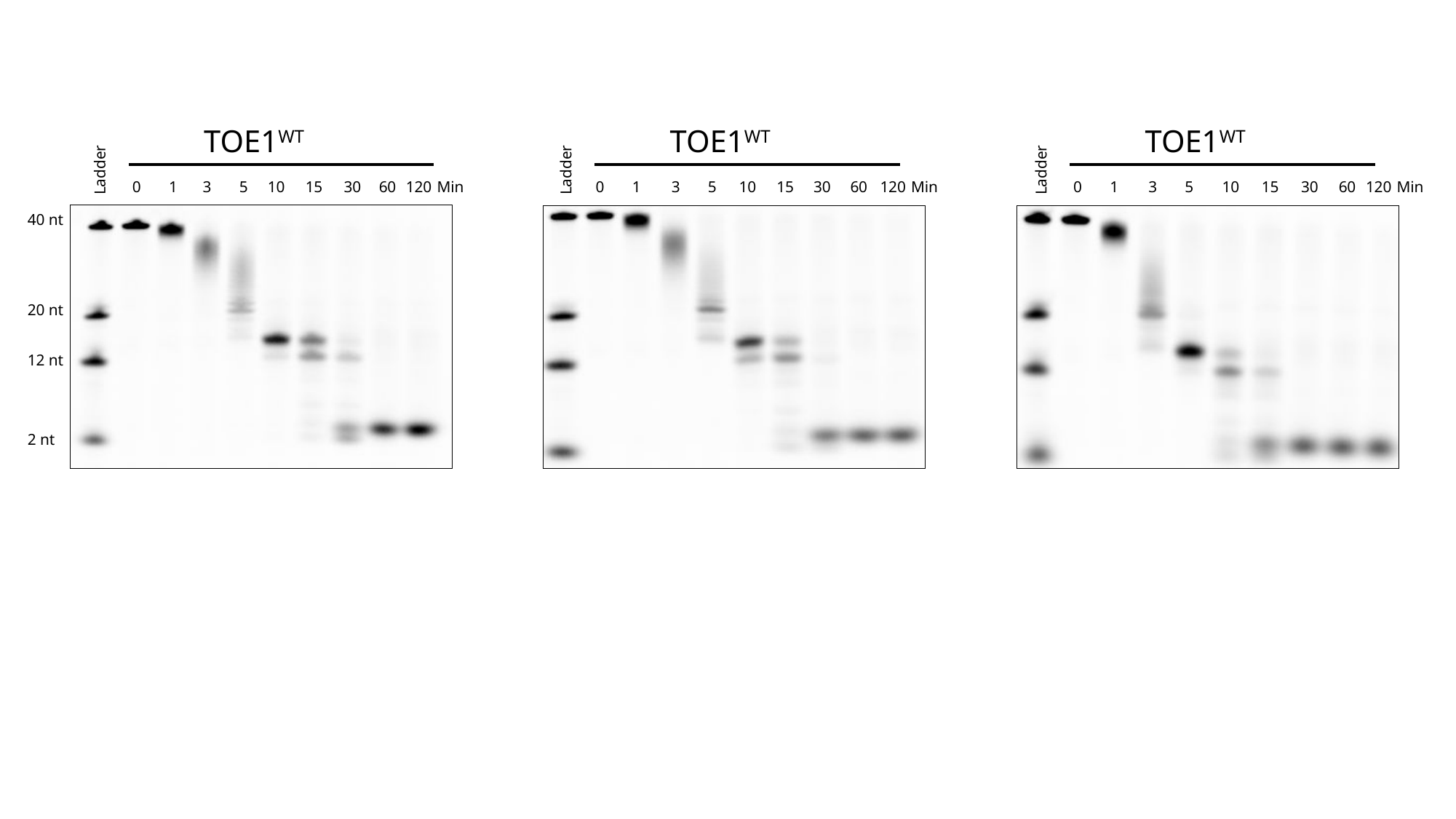

TOE1WT
TOE1WT
TOE1WT
Ladder
Ladder
Ladder
0
1
3
5
10
15
30
60
120
Min
0
1
3
5
10
15
30
60
120
Min
0
1
3
5
10
15
30
60
120
Min
40 nt
20 nt
12 nt
2 nt

## Slide 3
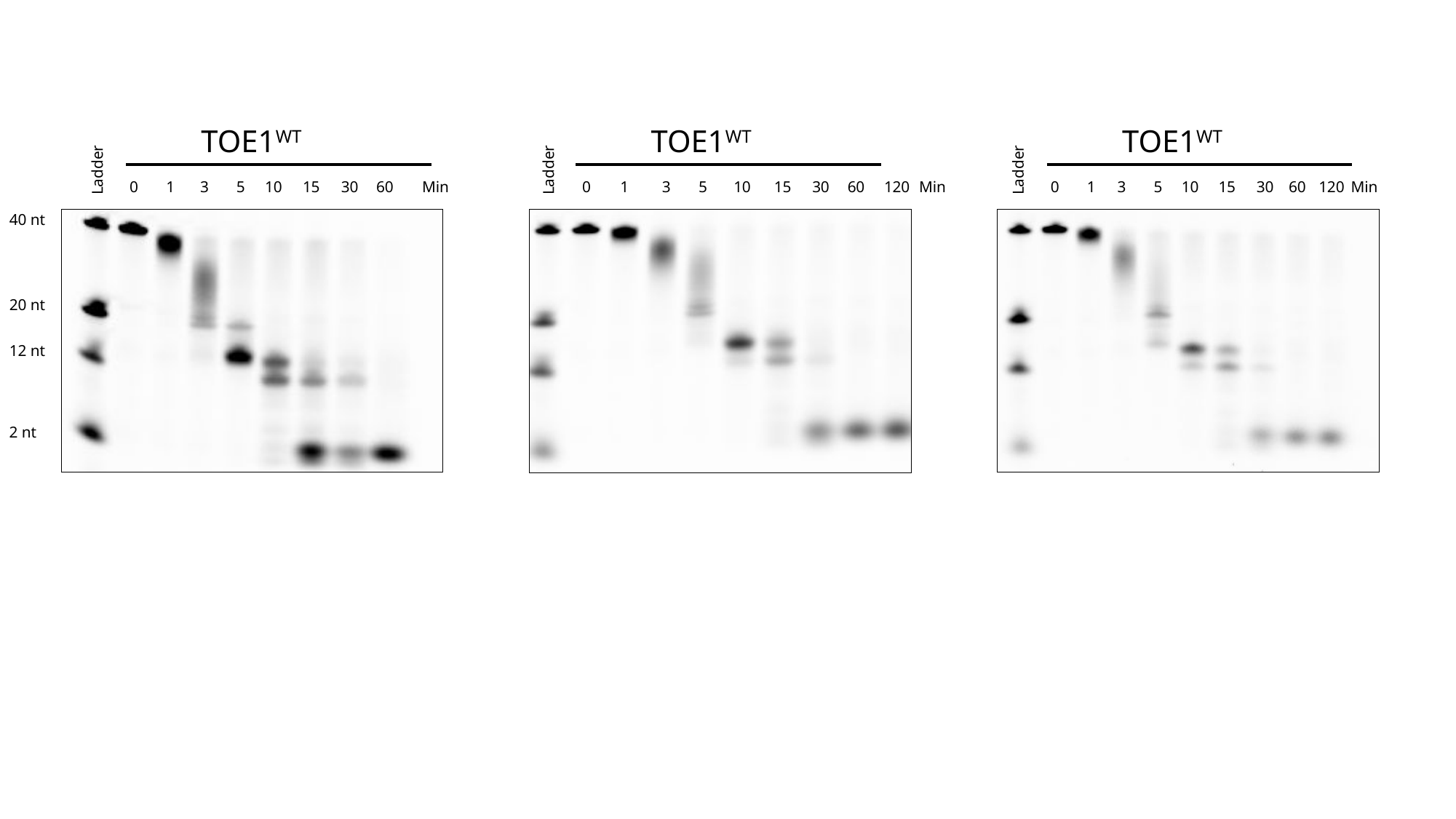

TOE1WT
TOE1WT
TOE1WT
Ladder
Ladder
Ladder
0
1
3
5
10
15
30
60
Min
0
1
3
5
10
15
30
60
120
Min
0
1
3
5
10
15
30
60
120
Min
40 nt
20 nt
12 nt
2 nt

## Slide 4
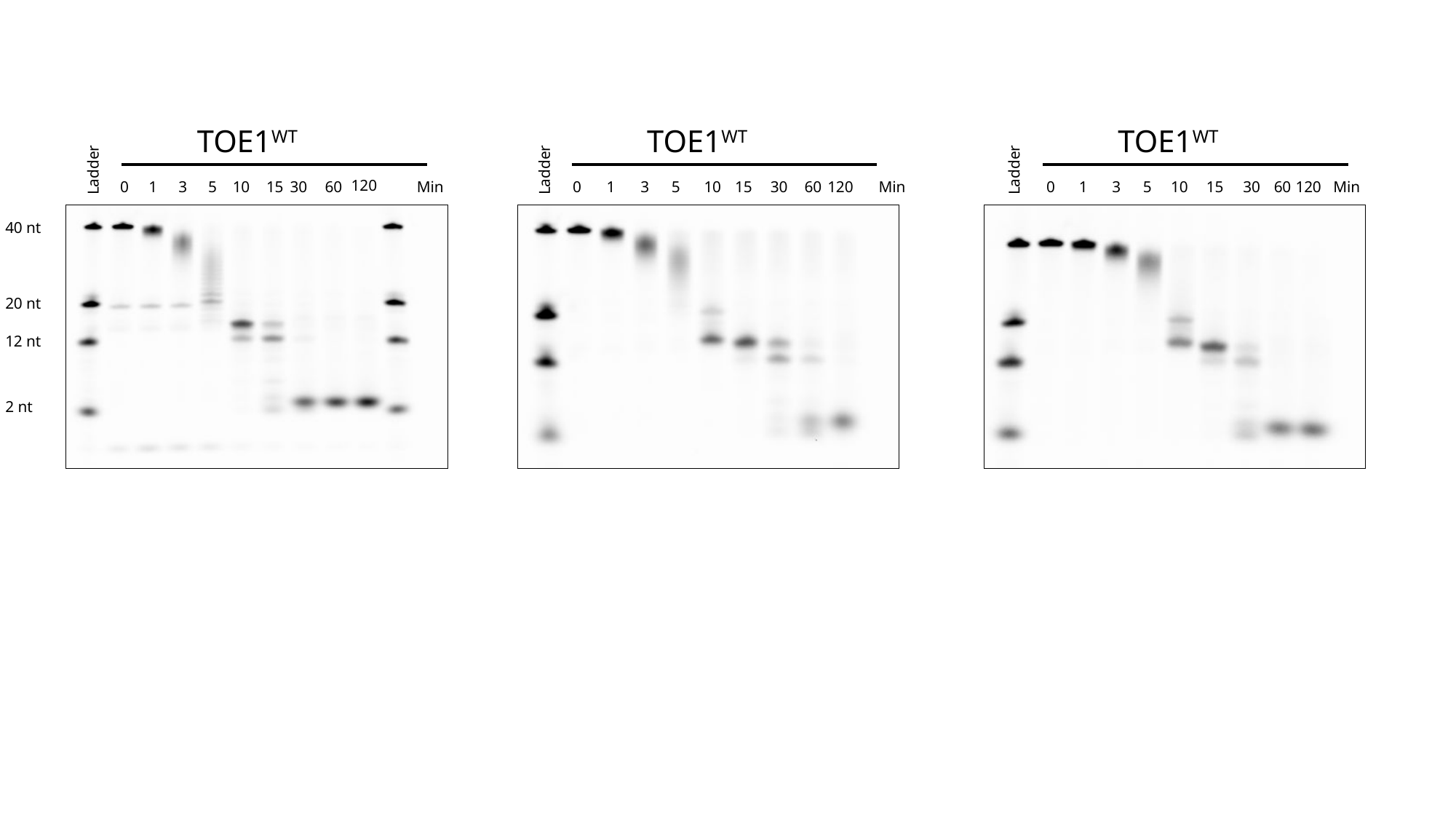

TOE1WT
TOE1WT
TOE1WT
Ladder
Ladder
Ladder
120
0
1
3
5
10
15
30
60
Min
0
1
3
5
10
15
30
60
120
Min
0
1
3
5
10
15
30
60
120
Min
40 nt
20 nt
12 nt
2 nt

## Slide 5
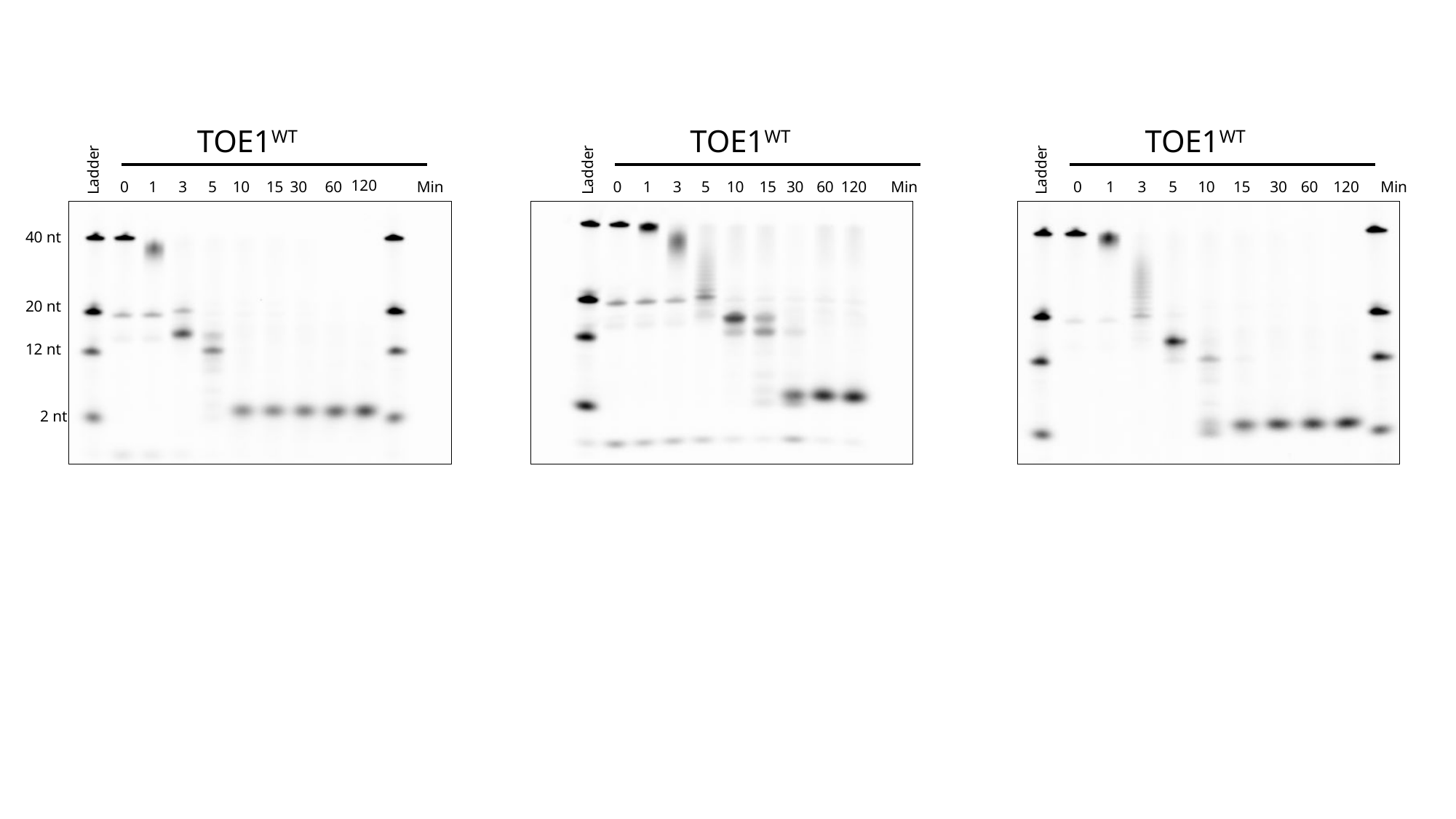

TOE1WT
TOE1WT
TOE1WT
Ladder
Ladder
Ladder
120
0
1
3
5
10
15
30
60
Min
0
1
3
5
10
15
30
60
120
Min
0
1
3
5
10
15
30
60
120
Min
40 nt
20 nt
12 nt
2 nt

## Slide 6
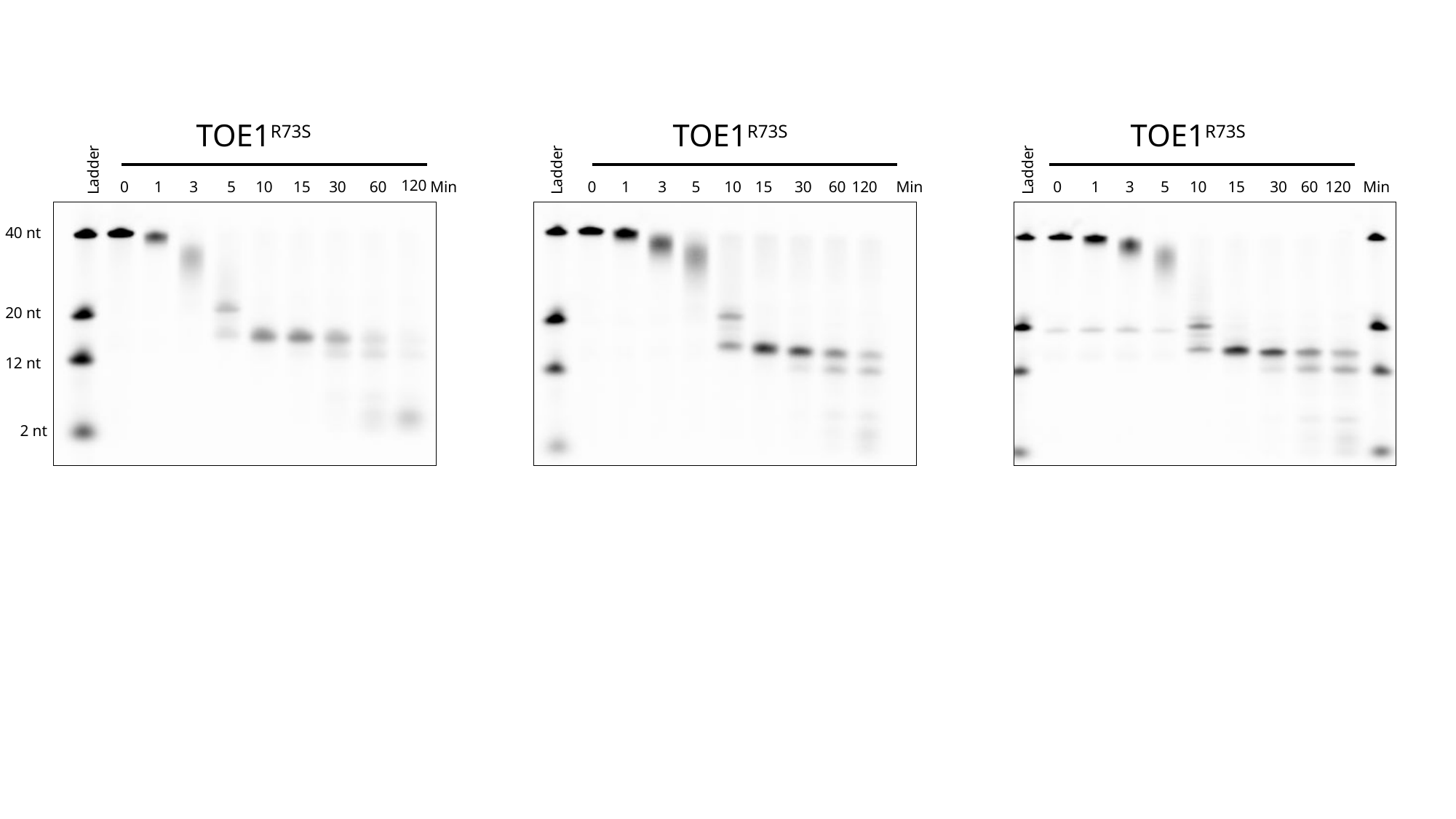

TOE1R73S
TOE1R73S
TOE1R73S
Ladder
Ladder
Ladder
120
0
1
3
5
10
15
30
60
Min
0
1
3
5
10
15
30
60
120
Min
0
1
3
5
10
15
30
60
120
Min
40 nt
20 nt
12 nt
2 nt

## Slide 7
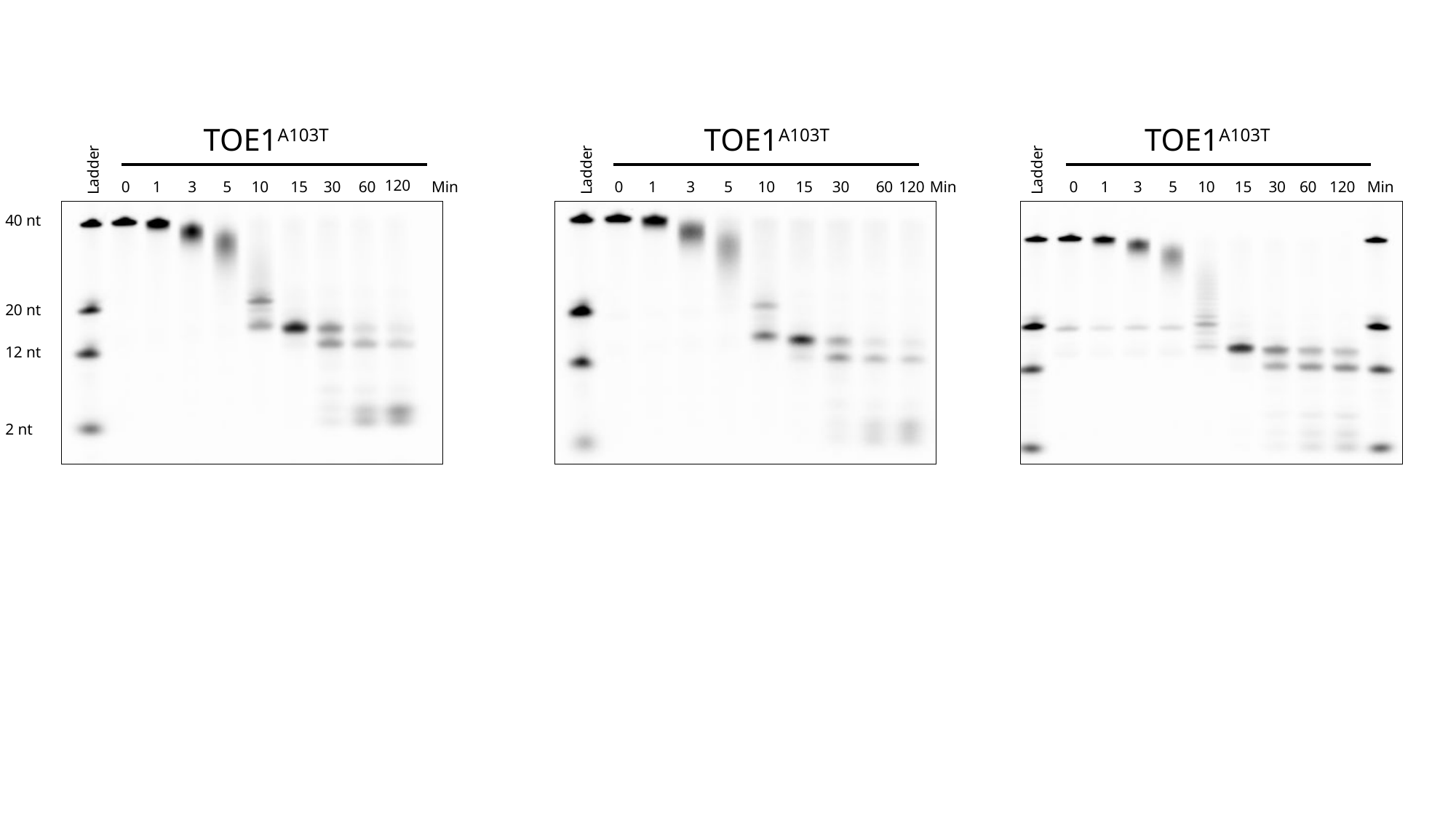

TOE1A103T
TOE1A103T
TOE1A103T
Ladder
Ladder
Ladder
120
0
1
3
5
10
15
30
60
Min
0
1
3
5
10
15
30
60
120
Min
0
1
3
5
10
15
30
60
120
Min
40 nt
20 nt
12 nt
2 nt

## Slide 8
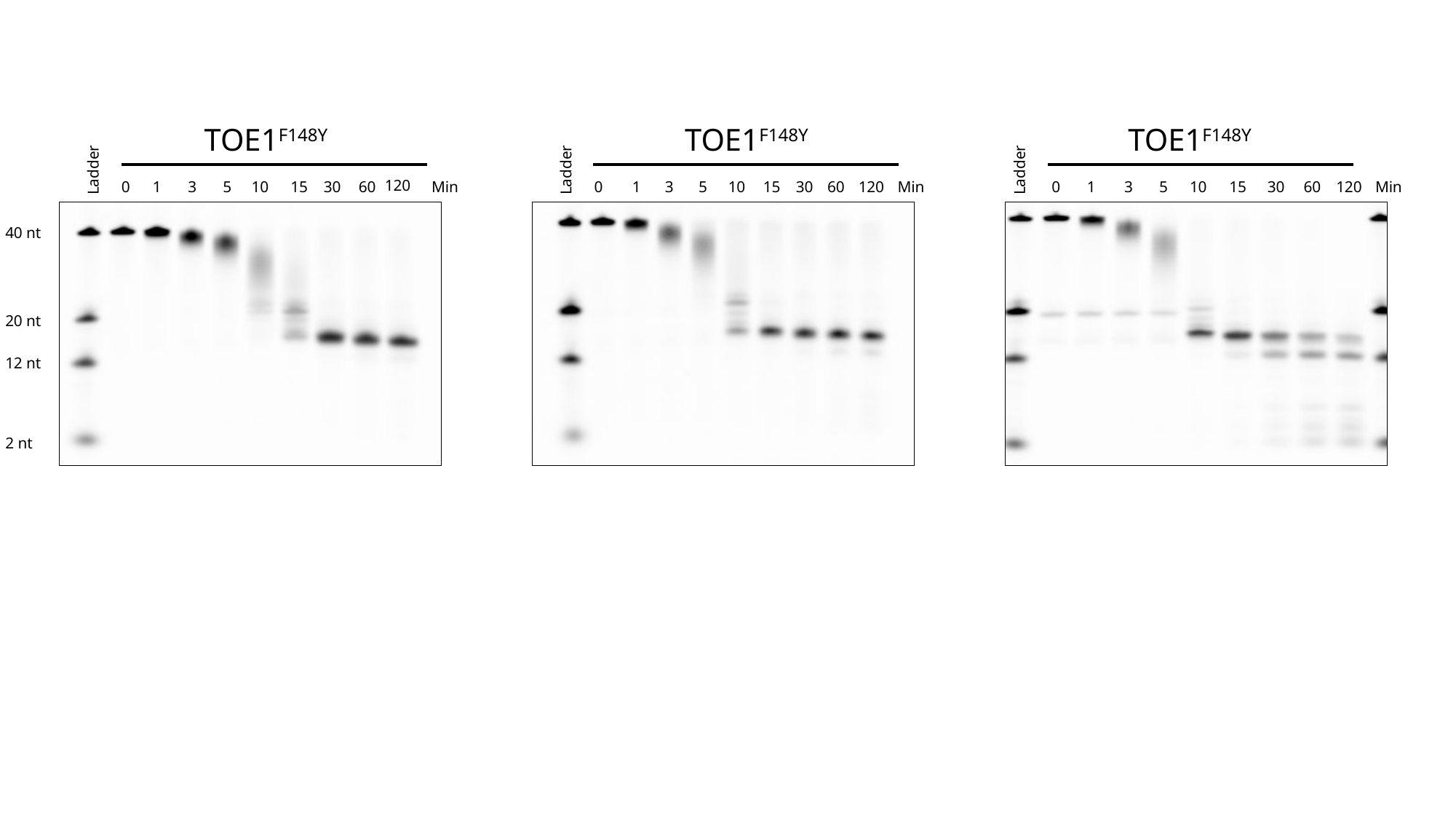

TOE1F148Y
TOE1F148Y
TOE1F148Y
Ladder
Ladder
Ladder
120
0
1
3
5
10
15
30
60
Min
0
1
3
5
10
15
30
60
120
Min
0
1
3
5
10
15
30
60
120
Min
40 nt
20 nt
12 nt
2 nt

## Slide 9
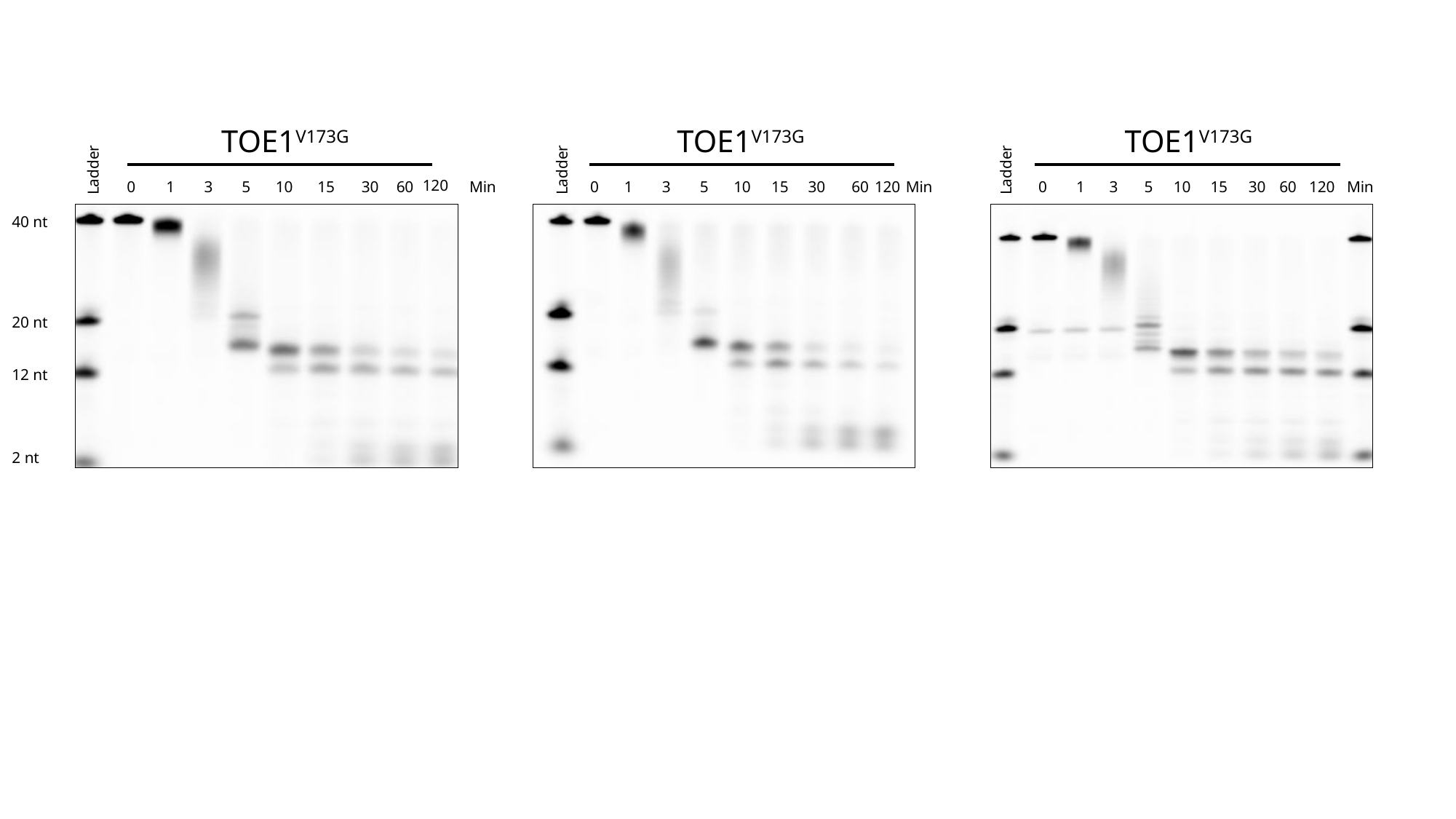

TOE1V173G
TOE1V173G
TOE1V173G
Ladder
Ladder
Ladder
120
0
1
3
5
10
15
30
60
Min
0
1
3
5
10
15
30
60
120
Min
0
1
3
5
10
15
30
60
120
Min
40 nt
20 nt
12 nt
2 nt

## Slide 10
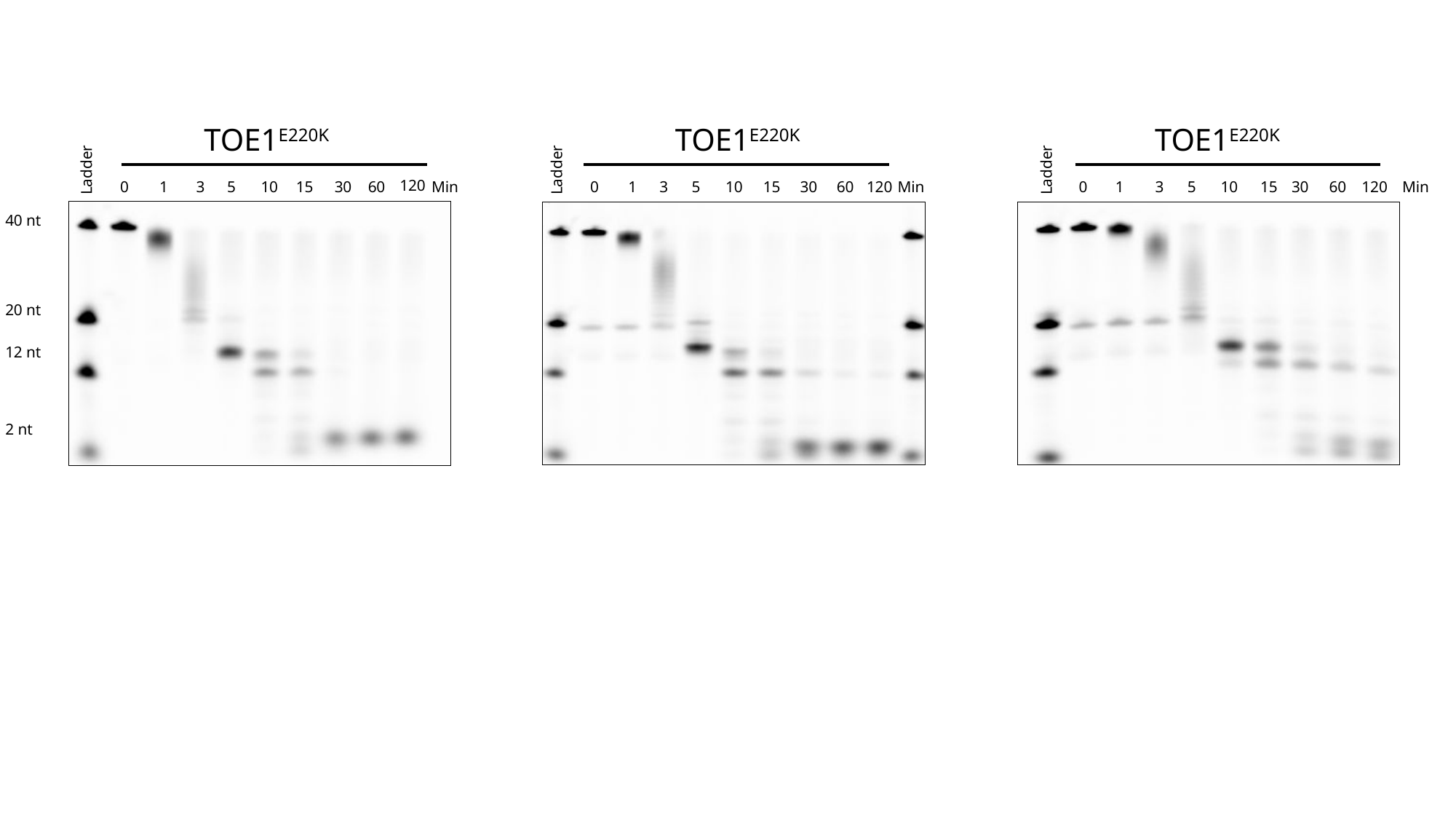

TOE1E220K
TOE1E220K
TOE1E220K
Ladder
Ladder
Ladder
120
0
1
3
5
10
15
30
60
Min
0
1
3
5
10
15
30
60
120
Min
0
1
3
5
10
15
30
60
120
Min
40 nt
20 nt
12 nt
2 nt

## Slide 11
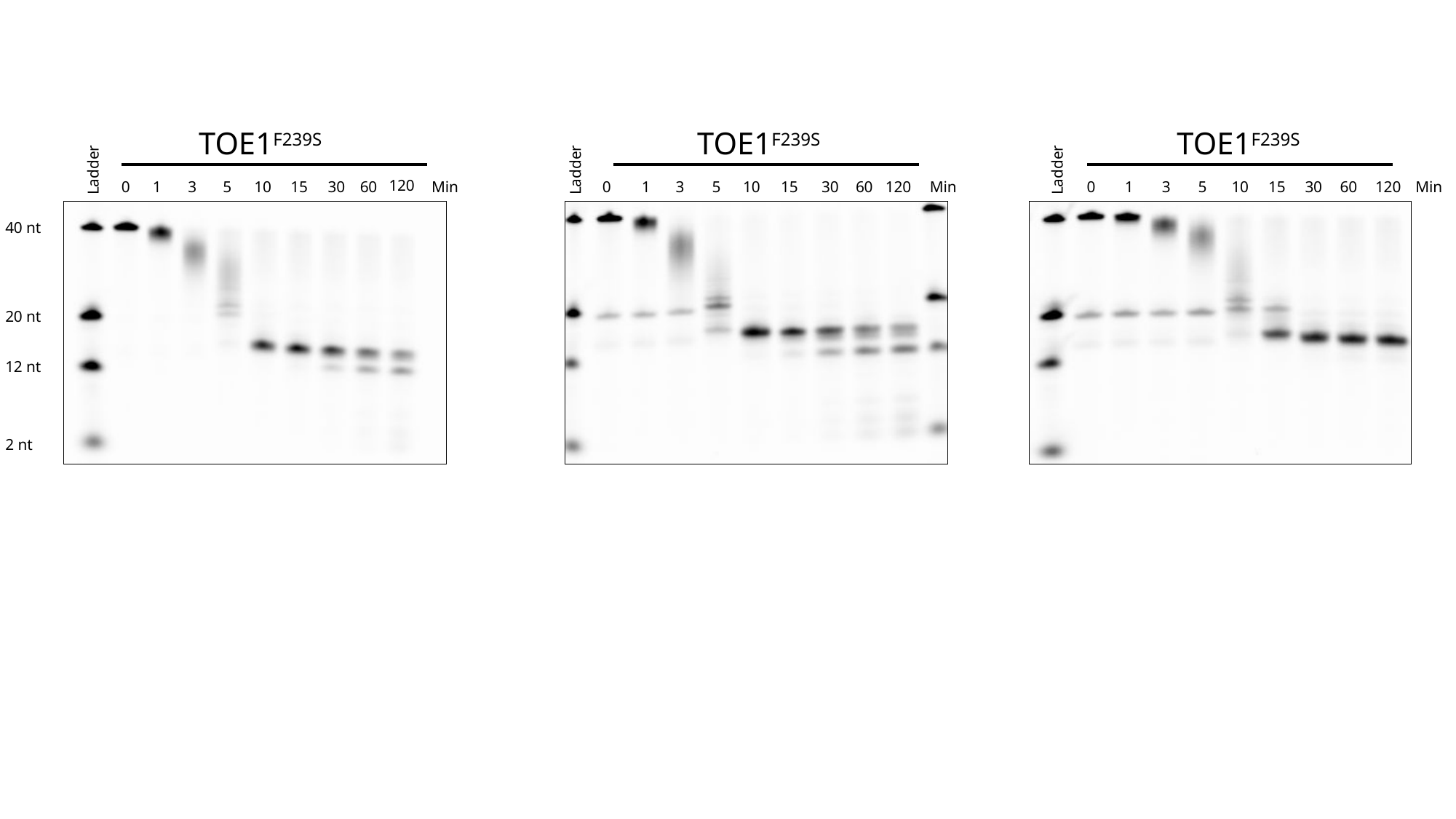

TOE1F239S
TOE1F239S
TOE1F239S
Ladder
Ladder
Ladder
120
0
1
3
5
10
15
30
60
Min
0
1
3
5
10
15
30
60
120
Min
0
1
3
5
10
15
30
60
120
Min
40 nt
20 nt
12 nt
2 nt

## Slide 12
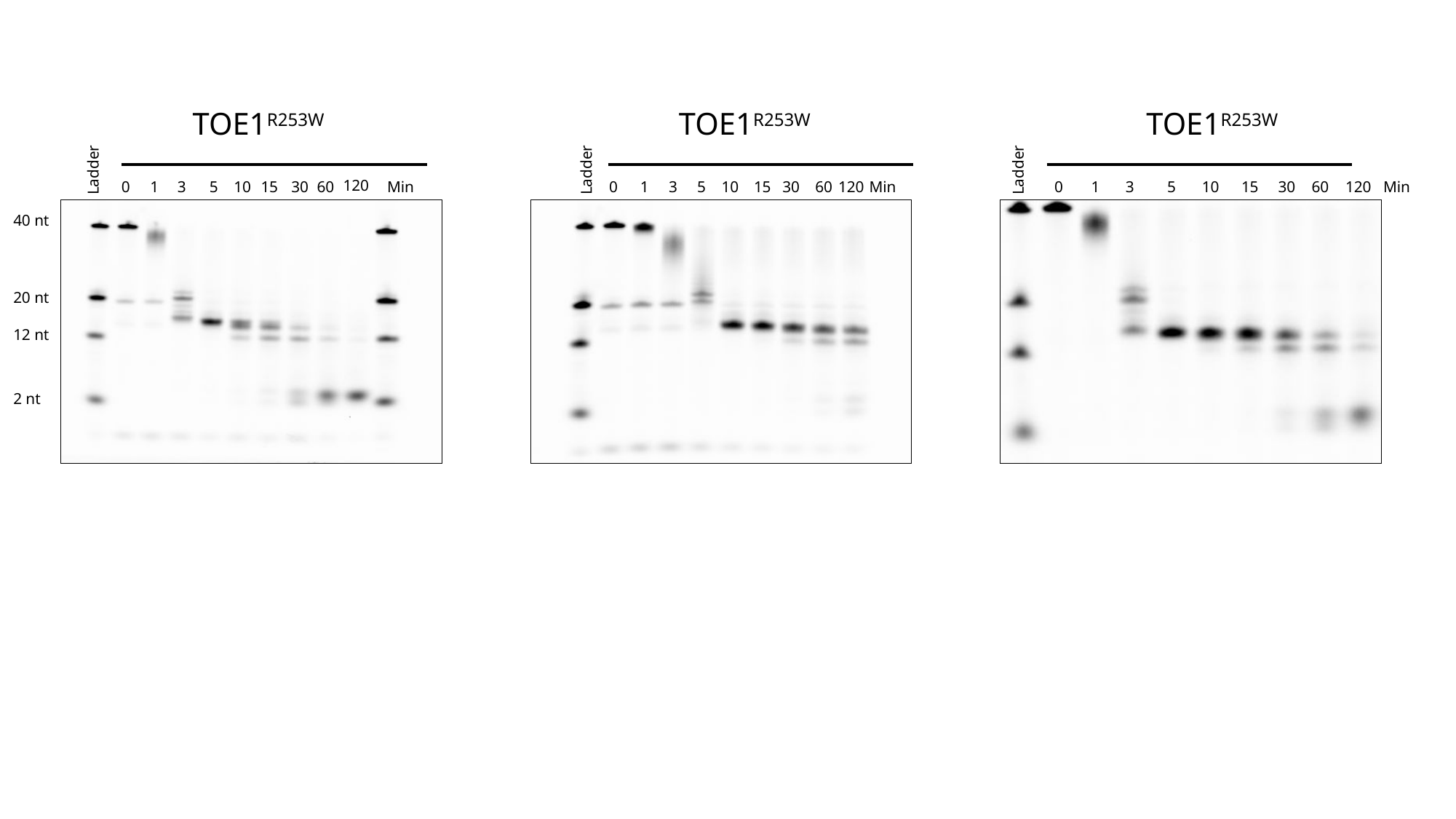

TOE1R253W
TOE1R253W
TOE1R253W
Ladder
Ladder
Ladder
120
0
1
3
5
10
15
30
60
Min
0
1
3
5
10
15
30
60
120
Min
0
1
3
5
10
15
30
60
120
Min
40 nt
20 nt
12 nt
2 nt

## Slide 13
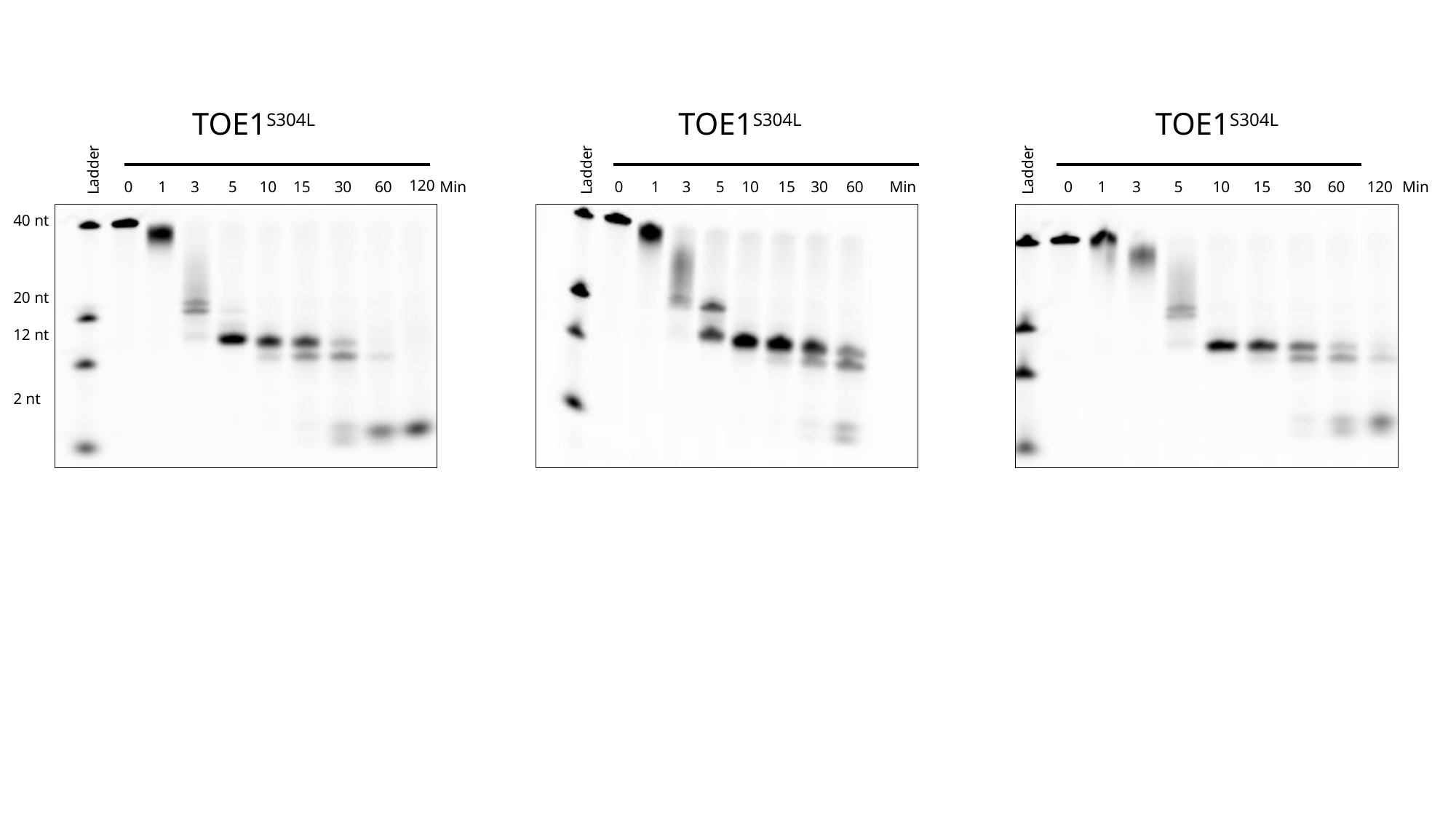

TOE1S304L
TOE1S304L
TOE1S304L
Ladder
Ladder
Ladder
120
0
1
3
5
10
15
30
60
Min
0
1
3
5
10
15
30
60
Min
0
1
3
5
10
15
30
60
120
Min
40 nt
20 nt
12 nt
2 nt

## Slide 14
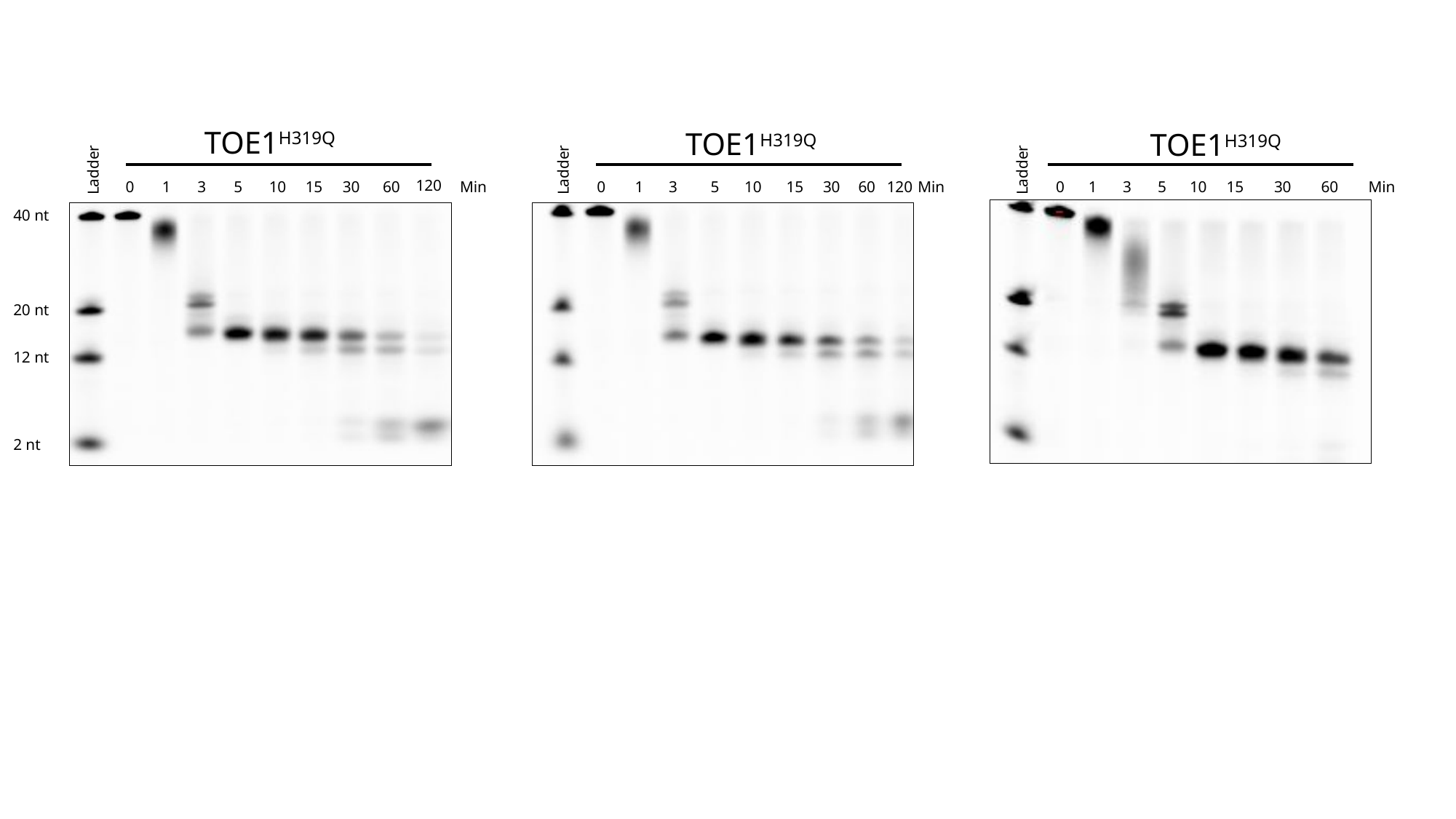

TOE1H319Q
TOE1H319Q
TOE1H319Q
Ladder
Ladder
Ladder
120
0
1
3
5
10
15
30
60
Min
0
1
3
5
10
15
30
60
120
Min
0
1
3
5
10
15
30
60
Min
40 nt
20 nt
12 nt
2 nt

## Slide 15
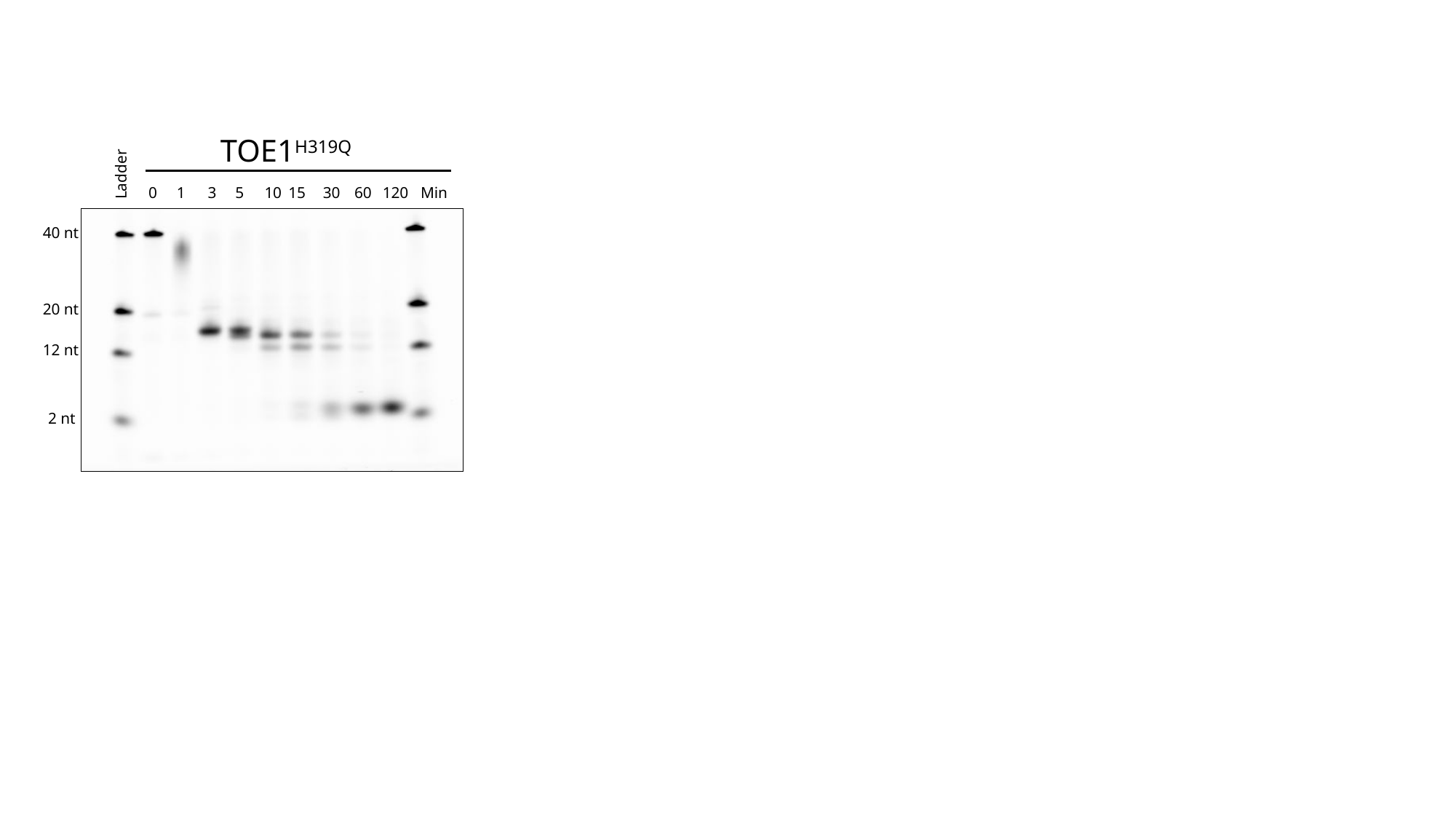

TOE1H319Q
Ladder
Min
10
15
30
60
120
5
3
1
0
40 nt
20 nt
12 nt
2 nt

## Slide 16
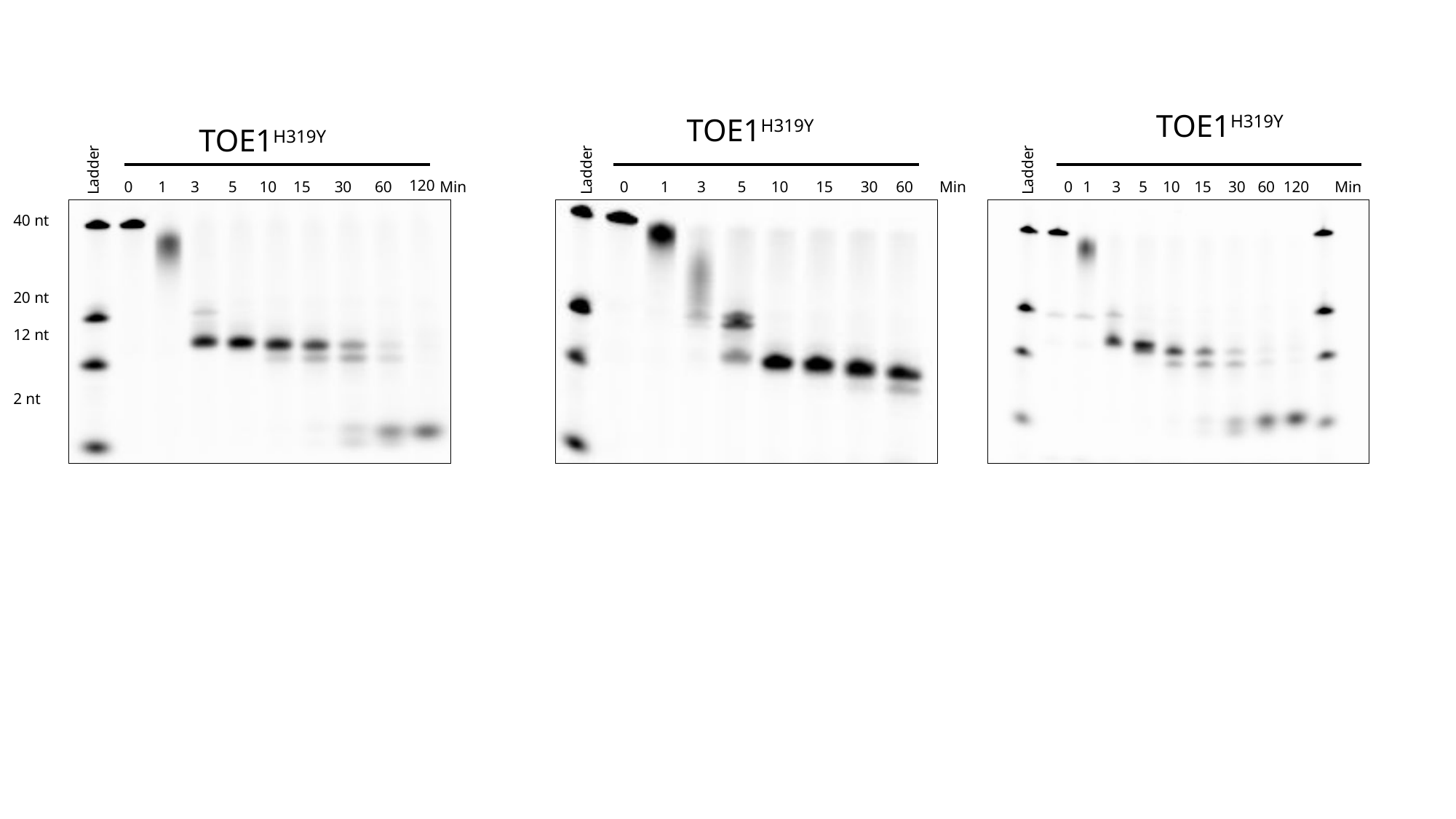

TOE1H319Y
TOE1H319Y
TOE1H319Y
Ladder
Ladder
Ladder
120
0
1
3
5
10
15
30
60
Min
0
1
3
5
10
15
30
60
Min
0
1
3
5
10
15
30
60
120
Min
40 nt
20 nt
12 nt
2 nt

## Slide 17
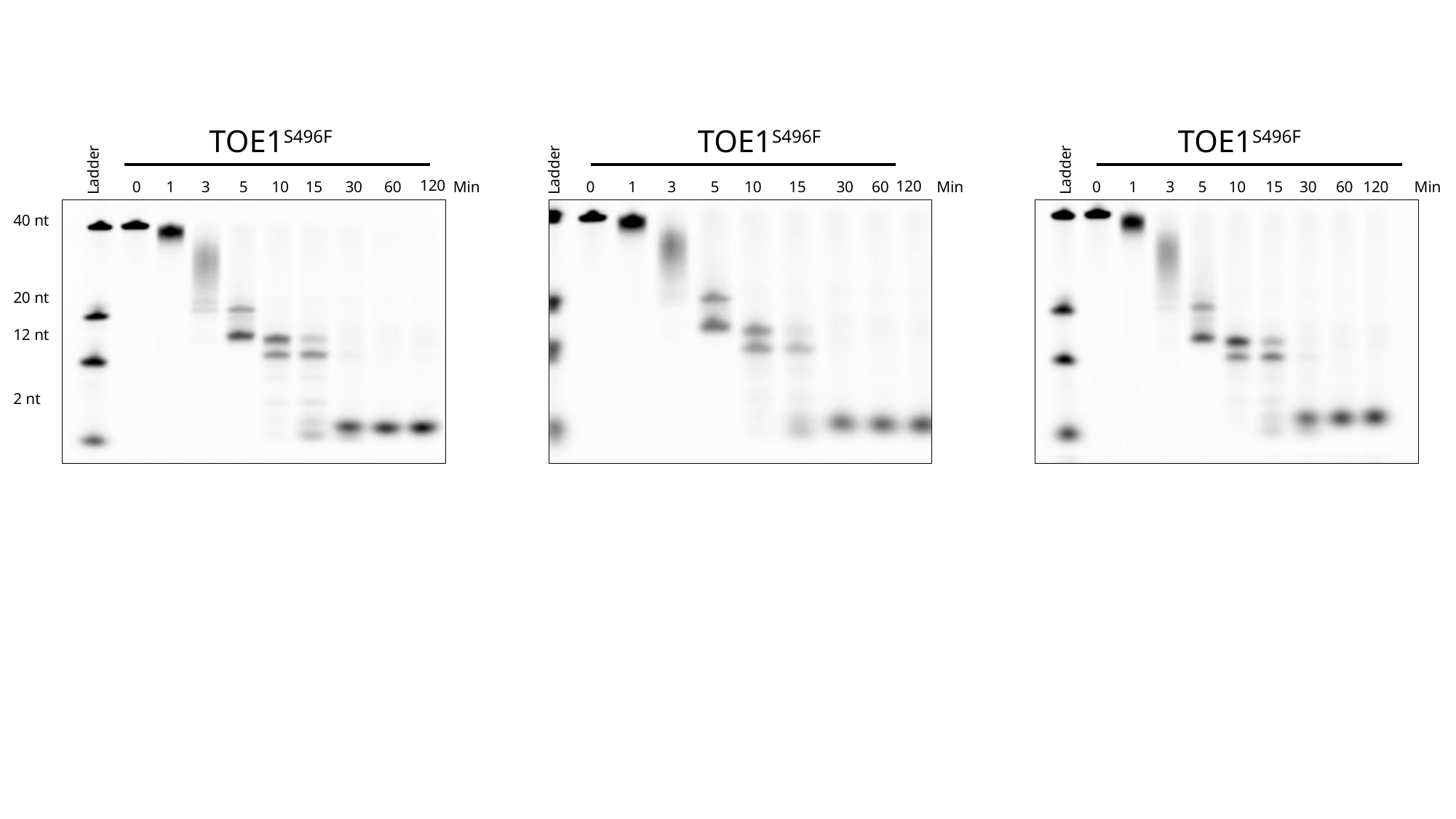

TOE1S496F
TOE1S496F
TOE1S496F
Ladder
Ladder
Ladder
120
120
0
1
3
5
10
15
30
60
Min
0
1
3
5
10
15
30
60
Min
0
1
3
5
10
15
30
60
120
Min
40 nt
20 nt
12 nt
2 nt

## Slide 18
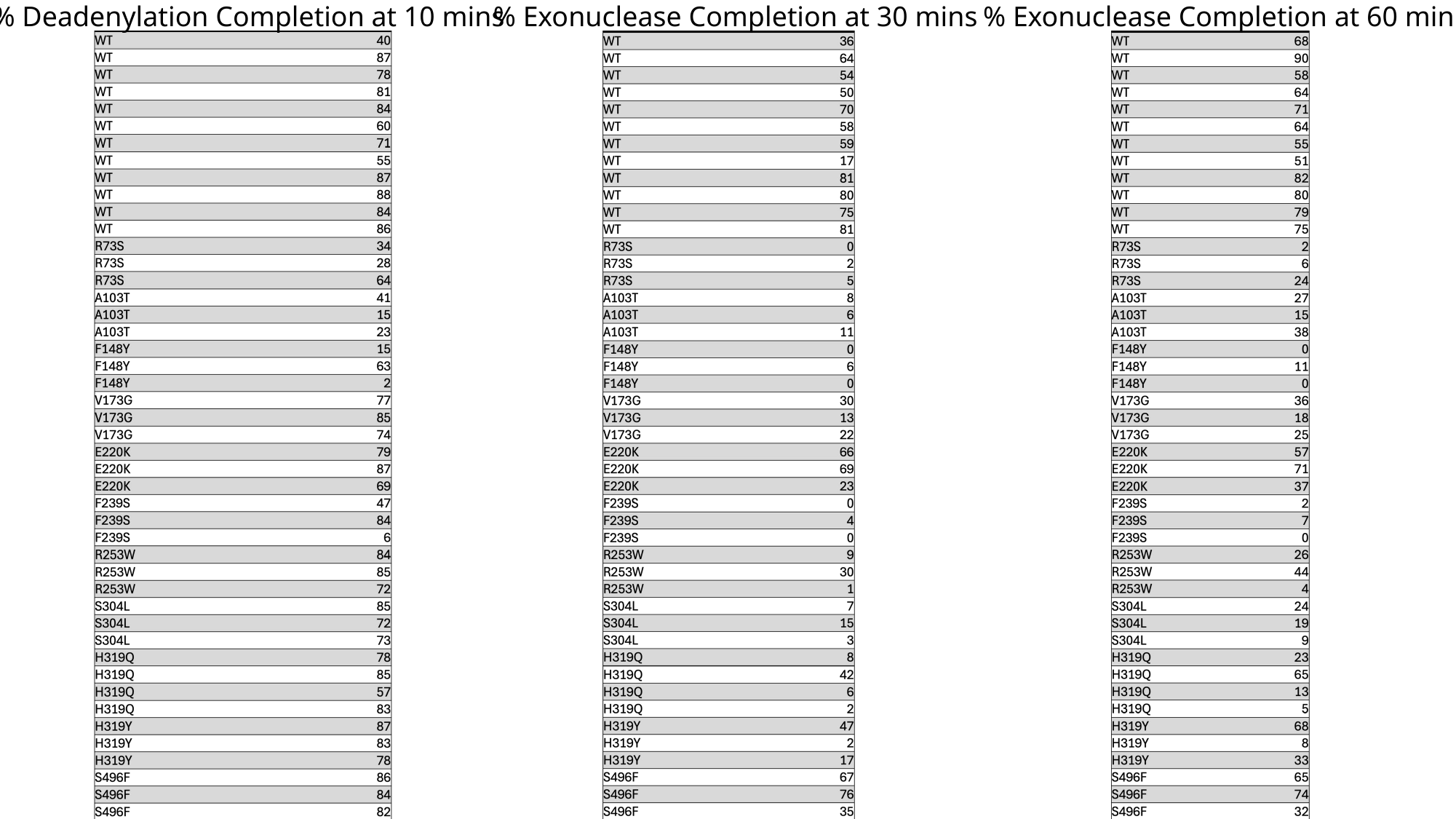

% Deadenylation Completion at 10 mins
% Exonuclease Completion at 30 mins
% Exonuclease Completion at 60 mins
